# EARLY-ALS: a multicentre study on prodromal amyotrophic lateral sclerosis

**DOI:** 10.1016/j.ebiom.2026.106505

**Published:** 2026-10-09

**Authors:** Isabell Cordts, Ana Galhoz, Laura Tzeplaeff, Anne Gründel, Florian Kohlmayer, Andreas Schwersenz, Ines Marschalkowski, Bogdan Bjelica, Verena Panitz, Christian Schulze, Martin Svačina, Petra Rau, André C. Dorigan, Marcus Deschauer, Simon Witzel, David Brenner, Jochen H. Weishaupt, Sarah K. Bublitz, Stefan Lorenzl, Andreas Hermann, Tim Hagenacker, Joachim Wolf, Martin Regensburger, Helmar C. Lehmann, Maike F. Dohrn, Johannes Dorst, René Guenther, André Maier, Thomas Meyer, Daniel Zeller, Christoph Neuwirth, Markus Weber, Jan C. Koch, Matthias Boentert, Markus Weiler, Susanne Petri, Ute Weyen, Torsten Grehl, Michael P. Menden, Paul Lingor

**Affiliations:** a Department of Neurology, TUM University Hospital – Klinikum Rechts der Isar, TUM School of Medicine, Technical University of Munich, Munich 81675, Germany; b Computational Health Center, Helmholtz Munich, Neuherberg 85764, Germany; c Bitcare GmbH, Munich 81675, Germany; d TUM ForTe, Munich 80333, Germany; e Center for ALS and Other Motor Neuron Disorders, Department of Neurology, Alfried Krupp Krankenhaus, Essen 45131, Germany; f Department of Neurology, Hannover Medical School, Hannover 30625, Germany; g PRACTIS Clinician Scientist Program, Dean’s Office for Academic Career Development, Hannover Medical School, Hannover 30625, Germany; h Department of Neurology, Heidelberg University Hospital, and Faculty of Medicine, Heidelberg University, Heidelberg 69120, Germany; i Department of Neurology, Medical Faculty of the RWTH Aachen University, Aachen 52074, Germany; j Department of Neurology, Faculty of Medicine and University Hospital Cologne, University of Cologne, Cologne 50937, Germany; k Philipps University Marburg & Department of Neurology, University Hospital Gießen and Marburg, Marburg 35043, Germany; l Department of Neurology, University of Ulm, Ulm 89081, Germany; m Division of Neurodegeneration, Department of Neurology, Mannheim Center for Translational Neurosciences, Medical Faculty Mannheim, Heidelberg University, Mannheim 68167, Germany; n Department of Neurology and Palliative Care, Agatharied Hospital, Hausham 83734, Germany; o Institute of Palliative Care, Paracelsus Medical University, Salzburg 5020, Austria; p Translational Neurodegeneration Section “Albrecht Kossel”, and Center for Transdisciplinary Neurosciences Rostock (CTNR), University Medical Center Rostock, Rostock 18147, Germany; q German Center for Neurodegenerative Diseases (DZNE), Rostock/Greifswald, Rostock 18147, Germany; r Department of Neurology, Center for Translational Neuro- and Behavioral Sciences (C-TNBS), University Medicine Essen, Essen 45147, Germany; s Department of Neurology, Diako, Brüderklinikum Julia Lanz, Mannheim 68163, Germany; t Department of Molecular Neurology, FAU Erlangen-Nürnberg, Erlangen 91054, Germany; u Department of Neurology, Faculty of Medicine and University Hospital Carl Gustav Carus, TUD Dresden University of Technology, Dresden 01307, Germany; v Charité – Universitätsmedizin Berlin, Berlin 10117, Germany; w Department of Neurology, University Hospital Würzburg, Würzburg 97080, Germany; x HOCH, Kantonsspital St. Gallen, St. Gallen 9007, Switzerland; y Department of Neurology, University Medical Center Göttingen, Göttingen 37075, Germany; z University of Münster, Münster 48149, Germany; aa Neuromedical Centre, Department of Sleep Medicine, Klinikum Osnabrück, Osnabrück 49074, Germany; ab BG University Hospital Bochum, Bochum 44789, Germany; ac Department of Biochemistry and Pharmacology, Bio21 Molecular Science and Biotechnology Institute, The University of Melbourne, Parkville, VIC 3052, Australia; ad German Center for Neurodegenerative Diseases (DZNE), Site Munich, Munich 81377, Germany; ae Munich Cluster for Systems Neurology (SyNergy), Munich 81377, Germany

**Keywords:** Motor neuron disease, Early disease detection, Preclinical neurodegeneration, Environmental risk factors, Sex-specific differences, Case–control study

## Abstract

**Background:**

Neurodegenerative diseases often feature a prolonged phase of subtle changes preceding diagnosis. In amyotrophic lateral sclerosis (ALS), defining this prodromal period is critical for identifying early disease features and intervention windows.

**Methods:**

In this case–control study, 475 patients with ALS and 285 controls from 20 centres in Germany and Switzerland completed a digital questionnaire on prodromal symptoms and health-related factors. Logistic regression included subgroup analyses by sex and clinical strata.

**Findings:**

Patients with ALS reported a higher burden of prodromal complaints (OR 7.50, 95% CI 4.27–13.17; *P* < 0.001), particularly neuro-motor, sensory, and pain-related symptoms. Before symptom onset, patients consulted neurologists more often (OR 1.26, 1.10–1.44; *P* < 0.001) and females more often received speech therapy (OR 2.35, 1.05–5.28; *P* = 0.038). Prodromal herniated discs were more common in males (OR 2.21, 1.04–4.68; *P* = 0.038) and spinal-onset patients (OR 2.14, 1.25–3.67; *P* = 0.006). Patients more often had lower secondary education (OR 1.93, 1.24–3.01; *P* = 0.004), physically demanding occupations (OR 2.21, 1.42–3.43; *P* < 0.001), and higher consumption habits (*P* < 0.005). Weight trajectories differed by sex (*P* = 0.009), with pre-onset weight loss in male patients (*P* < 0.001).

**Interpretation:**

Patients with ALS retrospectively reported subtle motor impairment before recognised symptom onset, alongside altered healthcare use and sex- and subgroup-specific patterns. These findings suggest that sporadic ALS may be preceded by a prodromal period and identify mild motor impairment as a potential marker, pending prospective validation.

**Funding:**

DGM e. V.

**Research in context**

**Evidence before this study**

Prodromal phases are well established in several neurodegenerative diseases. In amyotrophic lateral sclerosis (ALS), early changes, including mild motor impairment (MMI), have mainly been described in genetic cohorts, whereas evidence in predominantly sporadic ALS remains limited. Prodromal symptoms, healthcare utilisation, comorbidities, lifestyle factors, and weight trajectories have not been systematically integrated within a single framework in predominantly sporadic ALS.

**Added value of this study**

In this multicentre case–control study of 475 patients with ALS and 285 controls, we identified patterns consistent with a prodromal period associated with MMI, increased neurological and speech therapy consultations, and sex- and subgroup-specific patterns. By integrating symptom profiles, healthcare interactions, environmental exposures, sociobiographical factors, and metabolic change, this study provides a comprehensive view of early ALS beyond purely genetic cohorts. Our findings support the concept of MMI in predominantly sporadic ALS and identify potentially actionable early signals for clinical recognition.

**Implications of all the available evidence**

Taken together with existing literature, our findings support the possibility that ALS is not an abrupt clinical event but evolves through a detectable prodromal phase. Recognition of early motor and metabolic changes, alongside patterns of healthcare utilisation, may facilitate earlier diagnosis and risk stratification. More broadly, systematic characterisation of prodromal features may help align ALS with other neurodegenerative diseases in which early-stage identification is increasingly central to therapeutic development.

## Introduction

Neurodegenerative diseases typically evolve through a presymptomatic phase before clinical diagnosis. This includes a clinically silent stage in which biomarkers may indicate disease presence (premanifest stage), followed by early symptoms that signal progression but remain insufficient for diagnosis (prodromal stage), and ultimately a clinically manifest stage defined by characteristic symptoms.^1^ Identifying these early stages is critical for understanding disease mechanisms and enabling therapeutic and preventive interventions while neuronal damage may still be limited.

Prodromal phases are well recognised in several neurodegenerative disorders. In Parkinson’s disease, features such as REM sleep behaviour disorder may precede motor symptoms by years and strongly predict disease.^2^ In Alzheimer’s disease, mild cognitive impairment defines the prodromal stage,^3^ while in Huntington’s disease, subtle motor, cognitive, or psychiatric changes can occur long before clinical diagnosis.^4^^,^^5^ In contrast, prodromal markers in amyotrophic lateral sclerosis (ALS) remain incompletely characterised.

Evidence, mostly from genetic cases, indicates that ALS is preceded by measurable prodromal changes. Early motor neuron dysfunction without disease-defining weakness has been reported, particularly in carriers of pathogenic genetic variants, including muscle cramps, reduced exercise tolerance, fasciculations, hyperreflexia, and electromyographic abnormalities, collectively referred to as mild motor impairment (MMI).^6^ Subtle cognitive and/or behavioural changes not meeting criteria for overt frontotemporal dementia (FTD), consistent with mild cognitive and/or behavioural impairment (MCI/MBI), may also precede overt muscle atrophy and weakness.^7^^,^^8^ Prodromal weight loss is increasingly recognised as an early feature of ALS, associated with poorer prognosis^9^, ^10^, ^11^ and hypothalamic atrophy, even in presymptomatic carriers of pathogenic variants.^12^ Despite these observations, non-motor symptoms remain poorly studied, underdiagnosed, and underreported in ALS, despite their substantial impact on quality of life, particularly during prodromal stages.^13^

ALS is increasingly considered a multistep disease in which several pathogenic processes accumulate over time before clinical onset.^14^ As most ALS cases are sporadic and genetic variants explain only part of disease heritability, cumulative lifetime environmental exposures and lifestyle factors, collectively termed the exposome, are likely to play an important role. In line with the gene-time-environment hypothesis, interaction between genetic susceptibility, ageing, and environmental exposures is thought to shape disease onset and progression.^15^ Understanding the ALS exposome is therefore critical as a potentially modifiable component of disease risk.

A wide range of environmental factors have been investigated in ALS, including occupational and environmental exposures (e.g., heavy metals, pesticides, air pollution, viruses), lifestyle-related factors (e.g., smoking, physical activity, dietary habits), and comorbid medical conditions (e.g., metabolic disorders, cancer, neuroinflammation), yet most reported associations lack consistent or convincing evidence.^16^, ^17^, ^18^ A meta-analysis identified five environmental factors including heavy metals, pesticides, physical trauma, electric shock, and organic solvents as being associated with ALS onset.^19^ Nevertheless, research in this field remains challenging, as environmental exposures are heterogeneous, often subtle, and accumulate over decades, making them difficult to quantify and posing substantial methodological limitations for epidemiological studies.^20^

In this multicentre study, we aimed to systematically assess self-reported symptoms, environmental exposures, and life events in the period preceding ALS onset. By comparing patients with ALS with controls largely drawn from the same environment, we enabled an integrated evaluation of individual symptoms alongside environmental and lifestyle factors within a single study framework.

## Methods

### Patient recruitment

This multicentre case–control study was conducted between September 2020 and March 2024 using an online questionnaire. Participants were aged ≥18 years and had a diagnosis of possible, probable, or definite ALS according to the revised El Escorial criteria,^21^ or a related ALS phenotype (Table 1). Patients were recruited at 20 specialised ALS clinics in Germany and Switzerland, mostly affiliated with the Network for Motor Neuron Diseases (MND-NET).^22^ Ethnicity was not systematically recorded as part of the study protocol. To minimise selection bias, all patients meeting the inclusion criteria and attending the ALS clinics during the recruitment period were identified as potential participants. Proxy-assisted completion was permitted for patients, who were not physically able to fill out the questionnaire themselves.

**Table 1:** Study group characteristics of patients with ALS and controls.

	ALS (n = 475)	Controls (n = 285)	*P*
Demographics			
Age [years], median (IQR)	63 (57, 70)	61 (54, 68)	0.002^a^
n.a., n (%)	79 (16.63)	137 (48.07)	
Sex, n (%)			
Female	180 (37.90)	168 (58.95)	<0.001^b^
Male	289 (60.84)	114 (40.00)	
n.a.	6 (1.26)	3 (1.05)	
Composition of control group			
Relationship to proband, n (%)			
Parents		20 (7.02)	
Siblings		10 (3.51)	
Children		31 (10.88)	
Uncle		1 (0.35)	
Partners		214 (75.09)	
Other		6 (2.11)	
n.a.		3 (1.05)	
Disease characteristics			
Age-at-onset			
Median (IQR), years	60 (53, 67)	–	
Early, n (%)	191 (40.21)	–	
Late, n (%)	185 (38.95)	–	
n.a., n (%)	99 (20.84)		
Site of onset, n (%)			
Spinal	378 (79.58)	–	
Bulbar	75 (15.79)	–	
n.a.	22 (4.63)		
ALSFRS-R, median (IQR)	33 (25, 38)	–	
Progression rate (ΔFRS)			
Median (IQR)	0.48 (0.26, 0.82)	–	
Slow, n (%)	153 (33.77)	–	
Moderate, n (%)	146 (32.23)	–	
Fast, n (%)	154 (34.00)	–	
n.a., n (%)	22 (4.63)		
Disease duration			
Median (IQR), months	29 (17, 50)	–	
Short, n (%)	228 (50.33)	–	
Long, n (%)	225 (49.67)	–	
n.a., n (%)	22 (4.63)		
Known pathogenic variant, n (%)			
*C9orf72*	9 (1.89)	–	
*SOD1*	4 (0.84)	–	
*TARDBP*	1 (0.21)	–	
*ANXA11*	1 (0.21)	–	
*TBK1*	1 (0.21)	–	
*SETX*	1 (0.21)	–	
n.a.	458 (96.42)		
ALS phenotype, n (%)			
Classical ALS (Charcot-type)	330 (69.47)	–	
Progressive bulbar palsy (PBP)	39 (8.21)	–	
Flail-arm syndrome	22 (4.63)	–	
Primary lateral sclerosis (PLS)	22 (4.63)	–	
Flail-leg syndrome	17 (3.58)	–	
Progressive muscular atrophy (PMA)	13 (2.74)	–	
ALS-FTD	4 (0.84)	–	
n.a.	28 (5.89)		

Continuous variables are presented as median and interquartile range (IQR), and categorical variables are reported as absolute numbers and percentages. Within the control group, the “Other” category included friends, caregivers, stepchildren and sons- or daughters-in-law. The progression rate was categorised into slow-, moderate-, and fast-progressors based on tertiles. Disease duration and age-at-onset were dichotomised at the median into short- and long-duration and early- and late-onset subgroups, respectively.

ALS, Amyotrophic lateral sclerosis; ALSFRS-R, Amyotrophic Lateral Sclerosis Functional Rating Scale—Revised; FTD, Frontotemporal dementia, n.a., not available.

a Mann–Whitney test.

b Fisher’s exact test.

Participants with another neurodegenerative disease, a neuroinflammatory disorder, or dementia were excluded. Controls consisted primarily of family members, partners, friends, or caregivers recruited either during study visits or via patient invitation. In the same setting, the questionnaire was also administered to patients with motoneuron diseases other than ALS, including spinal muscular atrophy (SMA), hereditary spastic paraplegia (HSP) and spinal and bulbar muscular atrophy (SBMA); analyses of these cohorts will be reported elsewhere. No formal sample size calculation was performed; the study aimed to include all eligible participants recruited at the participating centres during the predefined recruitment period.

### Data collection

The digital questionnaire was developed and conducted using the open-source survey software tool LimeSurvey (https://www.limesurvey.org). The questionnaire was designed de novo by the study investigators based on clinical expertise and literature review. Detailed information regarding questionnaire development and implementation is provided in the Supplementary Methods. Participants accessed the survey via a secure HTTPS connection using unique personal registration tokens provided during routine clinical visits. Both the questionnaire and completed responses were hosted on a secure web server, and patient questionnaires were matched to controls using these tokens. The digital platform enabled a personalised survey flow, with follow-up questions displayed conditionally based on previous responses. The survey comprised seven sections: 1) demographics; 2) disease-related aspects including self-reported ALS phenotype, genetic testing status and results, and medication; 3) prodromal complaints and early non-motor changes across organ systems; 4) healthcare system interactions; 5) comorbidities; 6) lifestyle changes including occupation, physical activity, diet, and social life; and 7) the self-assessed ALS Functional Rating Scale-Revised (ALSFRS-R). Most questions referred to the 10-year period preceding ALS symptom onset or, in controls, the 10 years prior to survey completion. The reference period was subdivided into four intervals (5–10 years, 1–5 years, 1–12 months, and <1 month). Most questions were closed-ended, with predefined response options, and several included an “Other” option allowing free-text input. One open-ended question in the prodromal complaints section (3) invited participants to describe any notable changes in the 10-year reference period that could not be explained by known comorbidities.

### Ethics

The study was approved by the Ethics Committee of TUM University Hospital (reference number 383/20 S) and by the ethics committees of all participating sites. Written informed consent was obtained from all participants prior to study participation. The study was conducted in accordance with the principles of the 2024 Declaration of Helsinki.

### Statistics

Data collected via LimeSurvey were exported to CSV format for analysis and comprised continuous, categorical, binary, and date-time variables. Date-time variables were converted into continuous measures by calculating time in years relative to the questionnaire’s reference date. Given the adaptive conditional logic of the survey and the instruction for participants to leave non-applicable items blank, missing values in binary variables were imputed as zero to mitigate data sparsity (details provided in the Supplementary Methods). In addition, continuous variables were normalised to account for scaling differences. Age data were missing for a substantial proportion of participants, particularly controls, owing to a data capture error affecting this variable alone. To avoid bias and loss of statistical power, age was therefore not included as a covariate in the primary analyses, as its inclusion would have required exclusion of these participants and markedly reduced sample size and statistical power. To assess the potential impact of age-related confounding, sensitivity analyses were performed excluding participants aged <40 years and, separately, within the subset of participants with available age data.

Univariate comparisons between patients with ALS and controls were performed using logistic regression, with statistical significance assessed using the Wald test. All analyses were stratified and adjusted by sex. Given the self-administered design and sparsity, analyses were conducted within an exploratory analytical framework, using a conventional *P*-value (*P*) threshold of *P* < 0.05. Effect sizes were reported as odds ratios (OR) with 95% confidence intervals (CI). False discovery rate (FDR) was controlled using the Benjamini–Hochberg method, and adjusted *P*-values (*P*_adj_) were reported when significance (*P* < 0.05) was observed. Complete lists of univariate results with both nominal and FDR-adjusted *P*-values are provided in the Supplementary Tables. Demographic and clinical group differences were additionally assessed using t-tests or Mann–Whitney tests for continuous variables, depending on distribution and sample size, and Fisher’s exact test for categorical variables.

Subgroup analyses were restricted to questionnaire items significantly enriched in patients with ALS in the primary statistical analyses and with sufficient data completeness. Analyses were stratified by sex, site-of-onset, progression rate, disease duration, and age-at-onset. Sex-specific effects were assessed using separate logistic regression models for males and females. Effects by site-of-onset, progression rate, disease duration, and age-at-onset were evaluated using multinomial logistic regression adjusted for sex with a multi-level outcome variable comprising controls and ALS subgroups, followed by pairwise contrasts. Site-of-onset was classified as bulbar-onset ALS or spinal-onset ALS. Progression rate was calculated as (48-ALSFRS-R)/disease duration, and patients were categorised into slow-, moderate-, and fast-progressors based on tertiles. Disease duration, defined as the number of months from the symptom onset to questionnaire completion, was dichotomised at the median into short- and long-duration subgroups. Age-at-onset was dichotomised at the median into early- and late-onset subgroups. Subgroup results were reported as OR with 95% CI and associated *P*-values.

Weight trajectories were analysed using linear mixed-effects models (LMMs) with time as a categorical variable and participant-specific random intercepts. Models included group (ALS vs. controls), sex, time, and their interactions to assess disease- and sex-specific effects.

All the analyses were performed in RStudio version 4.4.2.

### Role of funders

The study was funded by the Deutsche Gesellschaft für Muskelkranke e. V. (DGM). The funder had no role in study design, data collection, data analysis, data interpretation, or writing of the manuscript. The authors retained full responsibility for the content of the manuscript and for publication.

## Results

### Demographics and characteristics of the study groups

Demographic and clinical characteristics of the study groups in the EARLY-ALS multicentre case–control study were derived from 818 enrolled participants, including 513 patients with ALS and 305 controls; 58 participants (38 ALS and 20 controls) only partially completed the questionnaires and were excluded, resulting in a final analytic cohort of 760 participants, comprising 475 patients with ALS and 285 controls (Supplementary Fig. S1).

The cohort captures a broad demographic and clinical landscape of ALS spanning disease onset, phenotype, functional status, and genetic background (Table 1). In the ALS group, there were significantly more males. Significant differences in both sex (*P* < 0.001, Fisher’s exact test) and age (*P* = 0.002, Mann–Whitney test) were observed between patients with ALS and controls. The ALS symptom onset was reported with a median of 2 years (interquartile range [IQR]: 1–4 years) before questionnaire completion. Most patients with ALS reported a “classical ALS” phenotype (70%), while a minority presented with related phenotypic variants. A genetic cause of disease was reported by approximately 4% of patients with ALS.

### Questionnaire overview

Both the ALS and control group completed a core questionnaire covering seven categories. Including all main, sub-, and conditional items, 558 questions were presented, with patients with ALS answering an additional 52 disease-specific questions (Supplementary Tables S1 and S2). Excluding free-text responses yielded 491 questions for univariate analysis (Supplementary Fig. S2A), comprising 196 continuous, 191 binary, and 104 categorical variables. As the survey employed an adaptive conditional logic and non-applicable items were left blank by design, missing values in binary variables and questions concerning doctor visits were imputed as zero (see details in Supplementary Methods and Supplementary Fig. S2B).

### Prodromal complaints and non-motor changes

The ALS cohort reported significantly more prodromal changes prior to symptom onset compared with controls in response to an open-ended question (OR = 7.50, CI 4.27–13.17; *P* < 0.001; *P*_adj_ < 0.001). After exclusion of non-informative free-text entries, 117 patients with ALS (25%) and 15 controls (5%) reported 198 and 25 prodromal changes, respectively. Among patients with ALS who reported prodromal changes, 62% described two or more changes and 38% reported only one. Reported changes were manually categorised into 13 distinct symptom domains (Fig. 1A). Prodromal changes reported by patients with ALS were dominated by neuro/motor (57%) and sensory/vestibular (20%) symptoms (Fig. 1B and C). Within the neuro/motor domain, motor complaints predominated (76%), most commonly muscle weakness, followed by muscle cramps and gait disturbances. In the sensory/vestibular category, pain was most frequent (50%), particularly back pain, followed by muscle and limb pain. Notably, both neuro/motor and sensory/vestibular symptoms were commonly reported to have been present several years before ALS onset (defined based on symptoms in Supplementary Table S3), with median intervals of 1 year (IQR: 0–3) and 2 years (IQR: 1–7), respectively (Supplementary Fig. S3).

**Fig. 1:**
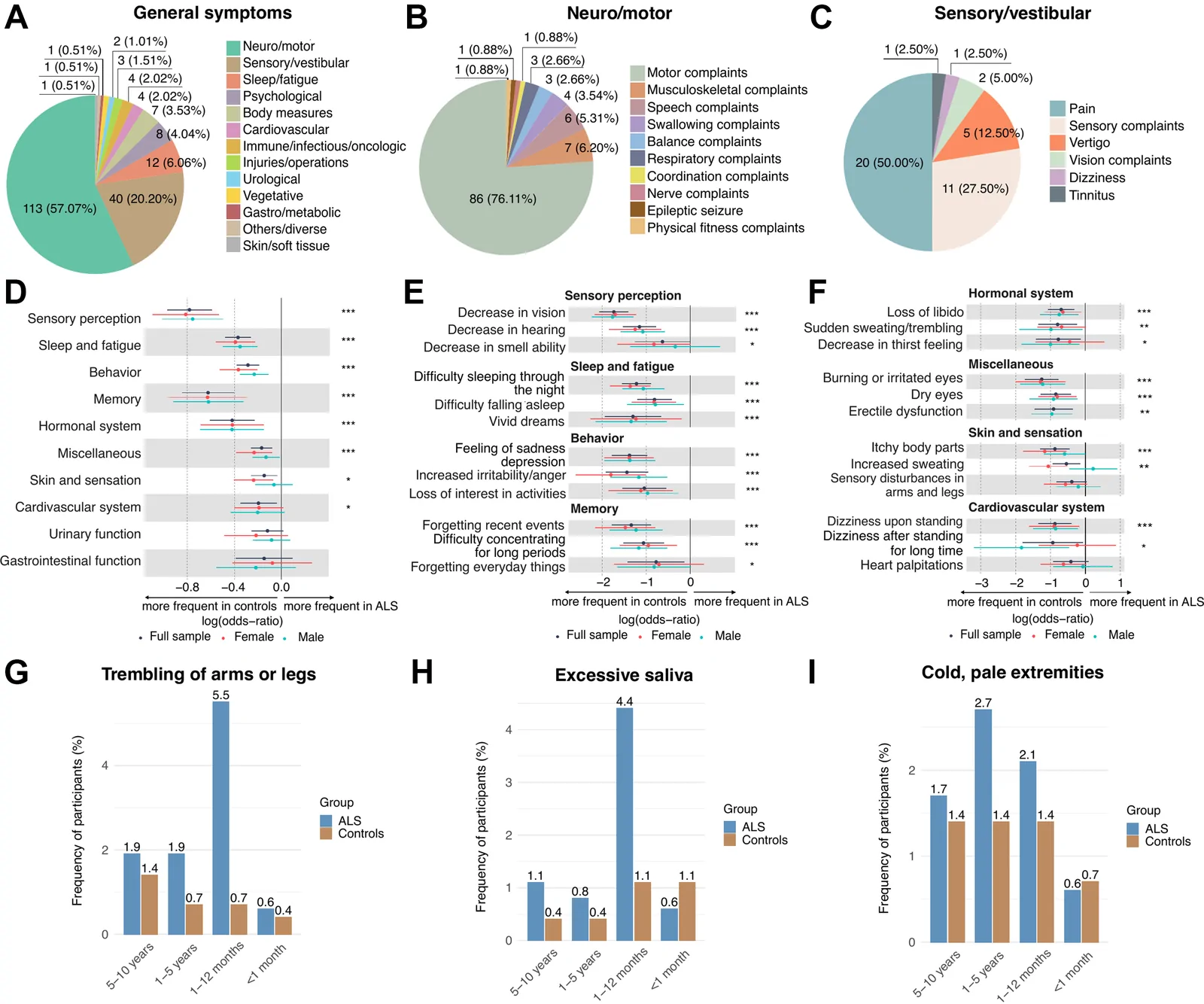
**Prodromal complaints of patients with ALS and control participants. (A–C)** Prodromal complaints provided by patients with ALS (n = 117) in response to an open-ended question. **(A)** Pie chart showing the relative frequencies of the 13 symptom domains into which the open-ended responses were manually categorised. Subcategories within the most frequently reported domains, **(B)** neuro/motor domain and **(C)** sensory/vestibular domain are visualised. **(D)** Forest plot for ten predefined categories of prodromal non-motor changes reported by patients with ALS and healthy individuals. **(E and F)** Forest plots of the eight significant prodromal non-motor changes (*P* < 0.05) and their top three subcategories (based on lowest *P*-values). In all forest plots, points to the left of the vertical axis indicate complaints more frequent in controls, and points to the right indicate complaints more frequent in patients with ALS. Within each forest plot, main categories are displayed in bold and followed by their respective subcategories. These reflect results from univariate analyses based on the full sample (ALS: n = 475; controls: n = 285), i.e., all participants adjusted for sex (black), and sex-stratified analyses (female in salmon, male in blue). The significance stars in the forest plots are based on nominal *P*-values of the full sample analysis, and indicated as *P* < 0.05 (∗), *P* < 0.01 (∗∗), *P* < 0.001 (∗∗∗) (logistic regression, Wald test). Error bars represent 95% confidence intervals. **(G–I)** Bar plots based on the full sample (ALS: n = 475; controls: n = 285) displaying the frequency of the three prodromal symptoms reported more frequently by patients with ALS than controls, originating from the miscellaneous and skin/sensation categories: **(G)** trembling of arms or legs, **(H)** excessive saliva, and **(I)** cold, pale extremities. The percentage of participants reporting each symptom is shown for four time intervals: 5–10 years, 1–5 years, 1–12 months, and <1 month before symptom onset (patients with ALS) or questionnaire completion (controls).

Prodromal non-motor changes assessed across predefined categories were reported more frequently by controls than by patients with ALS. Using 78 targeted questions across ten symptom categories (Fig. 1D), 40 symptoms were significantly more common in controls (OR<1; *P* < 0.05), whereas only one was enriched in patients with ALS (OR > 1; *P* < 0.05; Fig. 1D–F). Symptoms less frequently reported by patients with ALS clustered predominantly in the domains of sensory perception, sleep and fatigue, and behaviour; the most frequently reported symptoms within each of these domains are shown in Fig. 1E. Only a few non-motor symptoms were enriched in patients with ALS and clustered close to symptom onset. Trembling of the arms or legs was the most frequent prodromal complaint (OR = 3.24, CI 1.55–6.78; *P* = 0.002; *P*_adj_ = 0.011), while we observed a trend for excessive saliva (OR = 2.22, CI 1.00–4.95; *P* = 0.05), and cold, pale extremities (OR = 1.58, CI 0.82–3.03; *P* = 0.17). These symptoms were most commonly reported within 1–12 months before ALS onset (Fig. 1G–I).

### Healthcare interactions

Prodromal healthcare utilisation in ALS was characterised by increased neurology consultations. Over the 10 years preceding ALS symptom onset or survey completion, patients with ALS reported more frequent visits to neurologists than controls (OR = 1.26, CI 1.10–1.44; *P* < 0.001; *P*_adj_ = 0.007; Fig. 2A), with most visits occurring within 1–12 months before symptom onset (Fig. 2B). Visits to speech therapists were also more common in patients with ALS, particularly in women, but did not reach statistical significance when considering the full cohort (OR = 1.11, CI 0.99–1.25; *P* = 0.08; Fig. 2C). In contrast, controls more frequently consulted with specialists in oncology (OR = 0.89, CI 0.82–0.97; *P* = 0.011; *P*_adj_ = 0.048), dermatology (OR = 0.93, CI 0.87–0.99; *P* = 0.027; *P*_adj_ = 0.10) and endocrinology (OR = 0.92, CI 0.85–0.99; *P* = 0.032; *P*_adj_ = 0.12) (Fig. 2A).

**Fig. 2:**
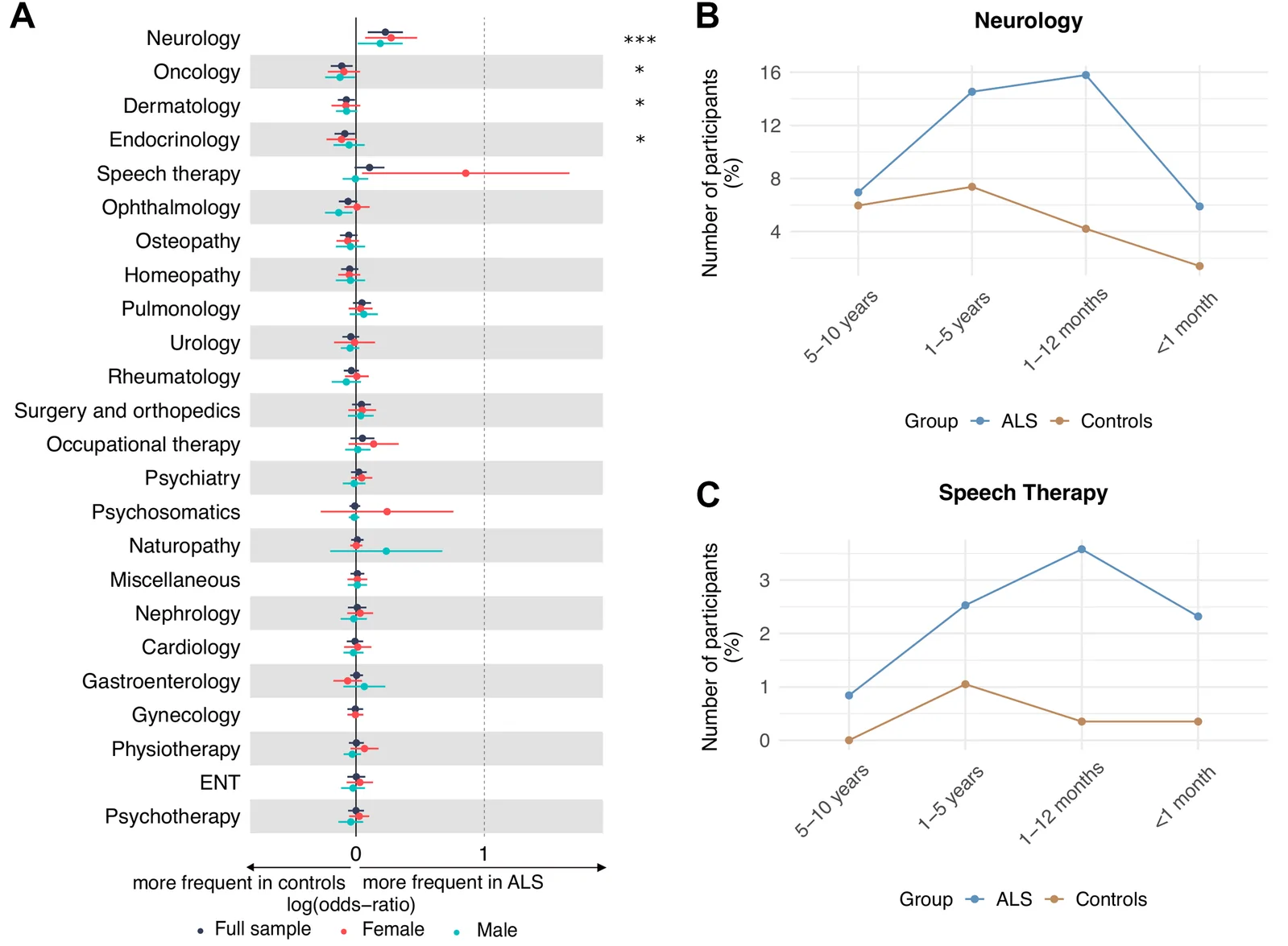
**Healthcare interactions of patients with ALS and control participants. (A)** Frequency of interactions with 24 different medical specialists reported by patients with ALS and controls. In the forest plot, points to the left of the vertical axis indicate specialists consulted more frequently by controls, and points to the right indicate specialists consulted more frequently by patients with ALS. Results illustrate univariate results based on the full sample (ALS: n = 475; controls: n = 285), i.e., all participants adjusted for sex (black), and sex-stratified analyses (female in salmon, male in blue). The significance stars are based on nominal *P*-values of the full sample analysis, and indicated as *P* < 0.05 (∗), *P* < 0.01 (∗∗), *P* < 0.001 (∗∗∗) (logistic regression, Wald test). Error bars represent 95% confidence intervals. **(B and C)** Percentage of participants who consulted neurologists and speech therapists, respectively, at four time intervals prior to symptom onset (patients with ALS) or questionnaire completion (controls). Percentages represent the number of participants who consulted each specialist during a specific period divided by the total number of participants (ALS: n = 475; controls: n = 285).

### Comorbidities

Musculoskeletal disorders prior to ALS symptom onset were more common in the ALS group (OR = 1.46, CI 1.04–2.05; *P* = 0.029; *P*_adj_ = 0.11; Fig. 3A). Within this category, herniated disc was the only condition reaching statistical significance, reported by 14% of patients with ALS compared with 7% of controls (OR = 1.85, CI 1.10–3.13; *P* = 0.021; *P*_adj_ = 0.09; Fig. 3B). We assessed whether this finding might reflect workplace physical load factors and found a negligible attenuation between the sex-adjusted model and the model additionally adjusting for intensive occupational physical activity (OR = 1.90, CI 1.13–3.30; *P* = 0.018). Of the patients with ALS reporting herniated disc, 40 (61%) provided timing information. Most events (68%) occurred 5 or more years prior to ALS symptom onset (Fig. 3C).

**Fig. 3:**
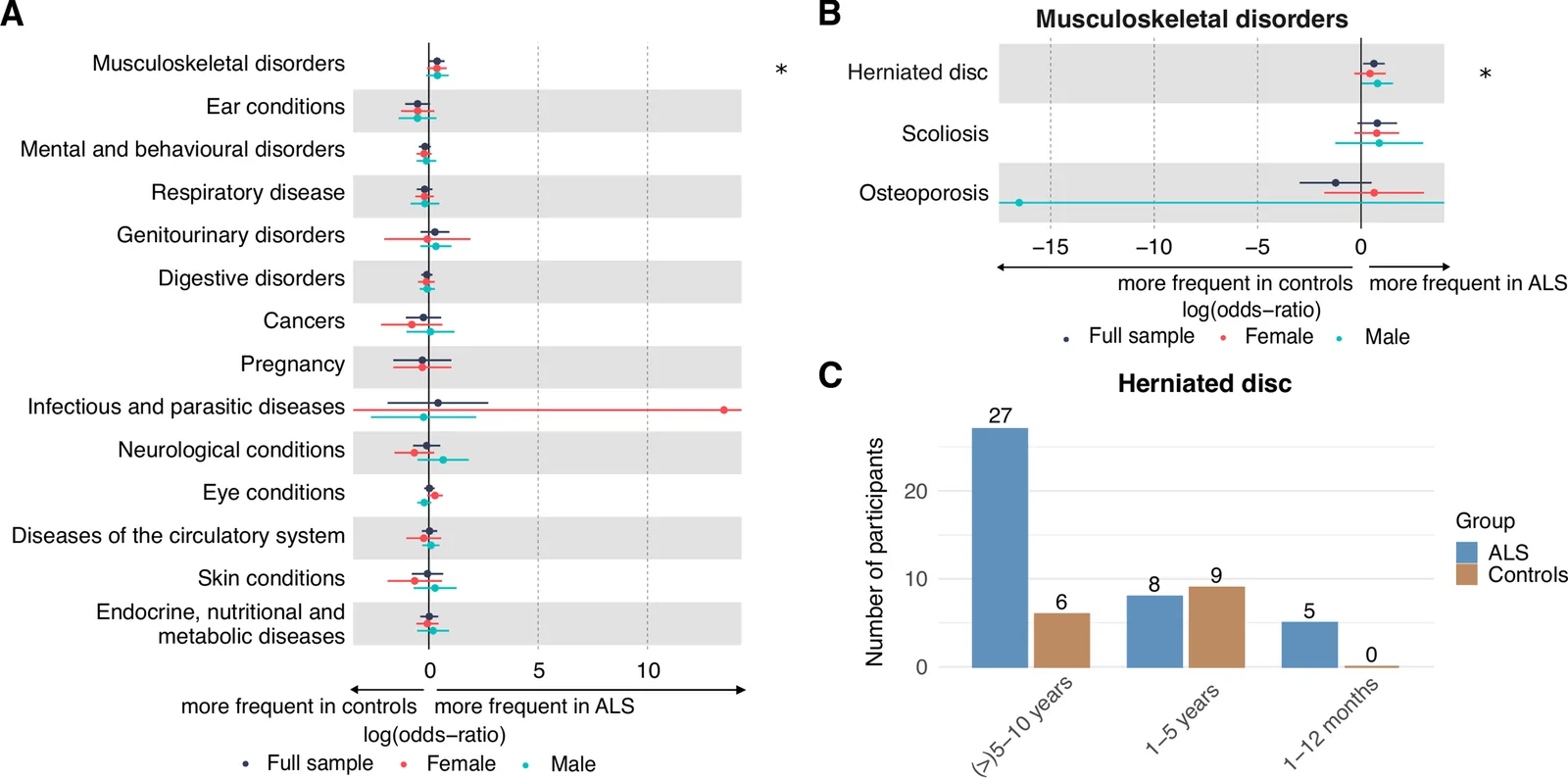
**Comorbidities in patients with ALS and control participants. (A)** Forest plot of univariate analyses comparing the frequency of 14 main categories of comorbidities between patients with ALS and healthy controls. While the original questionnaire assessed 15 comorbidity categories, those related to miscellaneous conditions were answered using free-text and were therefore excluded from this plot. **(B)** Visualisation of top three specific conditions within the musculoskeletal disorders category. In all forest plots in panels A and B, points to the left of the vertical axis indicate comorbidities more frequent in controls, and points to the right indicate comorbidities more frequent in patients with ALS. Results are from univariate analyses based on the full sample (ALS: n = 475; controls: n = 285), i.e., all participants adjusted for sex (black), and sex-stratified analyses (female in salmon, male in blue). *P*-values correspond to the full sample analysis and significance is indicated as *P* < 0.05 (∗), *P* < 0.01 (∗∗) (logistic regression, Wald test). Error bars represent 95% confidence intervals. **(C)** Distribution of herniated disc cases across time intervals: (>)5–10 years, 1–5 years and 1–12 months prior to symptom onset (patients with ALS) or prior to questionnaire completion (controls). Participants (ALS: n = 40; controls: n = 15) reported the exact date of onset for herniated disc diagnoses. For consistency, these dates were manually grouped into time intervals aligned with those used in other questions. Cases reported more than 10 years before symptom onset or questionnaire completion were included in the “(>)5–10 years” interval. Specifically, 22 of the 27 responses (ALS) and 5 of the 6 responses (controls) assigned to this interval referred to events occurring more than 10 years before symptom onset or questionnaire completion.

### Lifestyle changes and sociobiographical events

Social and life-event profiles differed between patients with ALS and controls. Patients with ALS were more likely to report being single (OR = 2.07, CI 1.26–3.39; *P* = 0.004; *P*_adj_ = 0.022), and to have children (OR = 1.41, CI 1.01-1.98; *P* = 0.045; *P*_adj_ = 0.16; Fig. 4A). In contrast, when major life events were considered collectively, controls reported significantly more personal and professional changes (OR = 0.31, CI 0.22–0.43; *P* < 0.001; *P*_adj_ < 0.001), largely accounted for by reports of serious illness affecting a close person (OR = 0.13, CI 0.09–0.18; *P* < 0.001; *P*_adj_ < 0.001), consistent with the predominance of relatives among controls (Fig. 4A, Table 1). Other major life events did not differ between groups (Supplementary Table S4).

**Fig. 4:**
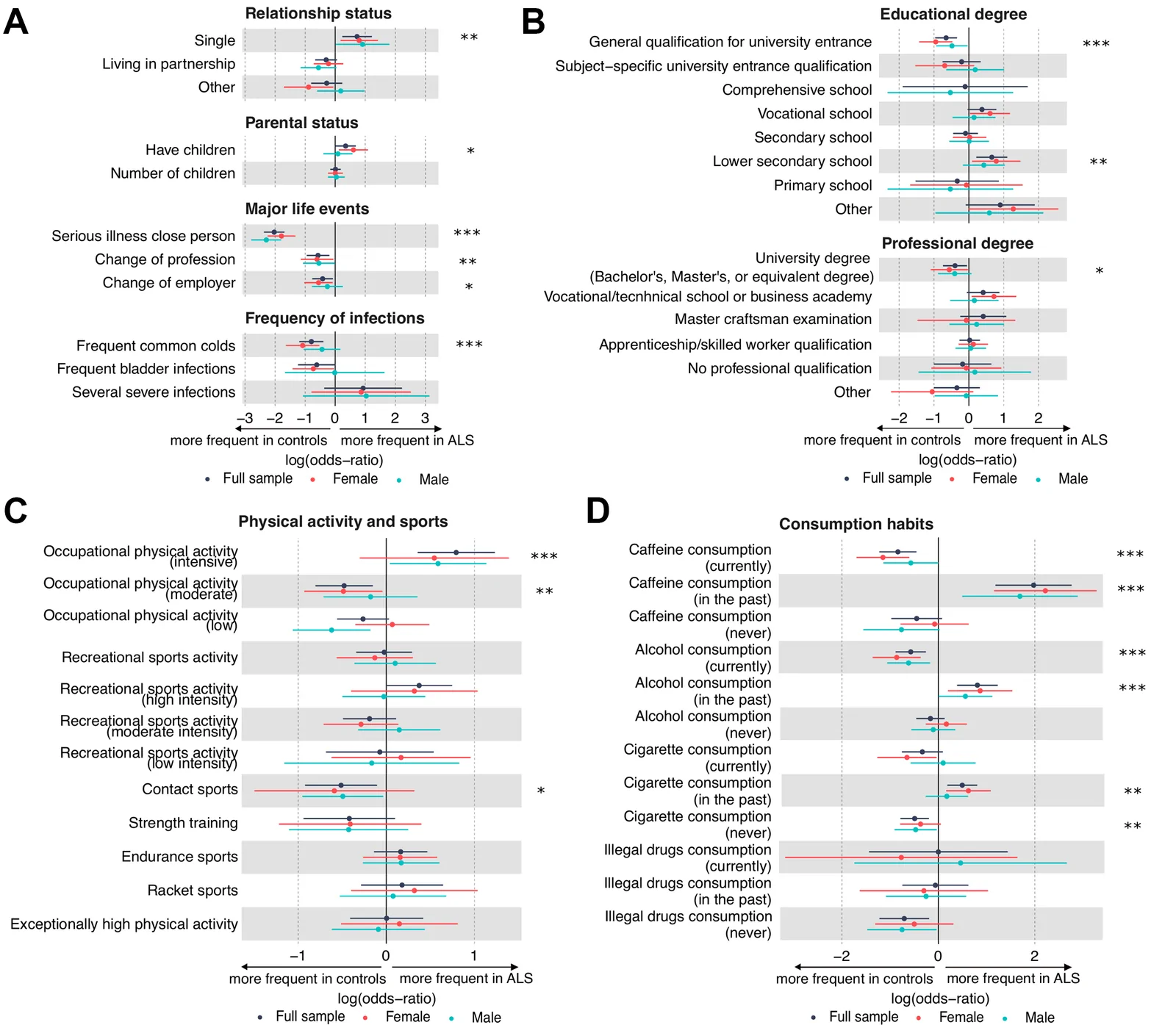
**Lifestyle differences between patients with ALS and control participants. (A)** Forest plots illustrating differences in relationship and parental status, major life events, and infection frequency. For life events and infections, only the top three subcategories (based on the lowest *P*-values) are displayed. **(B)** Distribution of educational and professional qualifications, presented in descending order where applicable. **(C)** Differences in physical activity, including work-related physical exertion (categorised from intensive to low), recreational engagement in sports, types of sports practised and the occurrence of episodes of exceptionally high physical activity (e.g., running a marathon). **(D)** Patterns of consumption habits, including caffeine, alcohol, and cigarette consumption, categorised by current use, past use, or never. In all forest plots, points to the left of the vertical axis indicate factors more frequent in controls, and points to the right indicate factors more frequent in patients with ALS. Within panels A, C, and D, main categories are displayed in bold and followed by their respective subcategories. All panels show univariate analyses results for the full sample (ALS: n = 475; controls: n = 285) adjusted for sex (black), and sex-stratified analyses (female in salmon, male in blue). Depicted significance is based on nominal *P*-values of the full sample analysis, and indicated as *P* < 0.05 (∗), *P* < 0.01 (∗∗), *P* < 0.001 (∗∗∗) (logistic regression, Wald test). Error bars represent 95% confidence intervals.

Infection-related experiences differed minimally between groups: only common colds were reported more frequently by controls (OR = 0.45, CI 0.30–0.67; *P* < 0.001; *P*_adj_ = 0.001) (Fig. 4A).

Educational attainment differed between patients with ALS and controls. Patients with ALS more frequently reported completion of lower secondary school (OR = 1.93, CI 1.24–3.01; *P* = 0.004; *P*_adj_ = 0.020), whereas controls more often reported holding a general qualification for university entrance (OR = 0.52, CI 0.38–0.71; *P* < 0.001; *P*_adj_ < 0.001) and university degrees (OR = 0.67, CI 0.48–0.95; *P* = 0.023; *P*_adj_ = 0.09) (Fig. 4B).

Occupational but not recreational physical activity differed between patients with ALS and controls. Patients with ALS were more likely to engage in physically demanding occupations (OR = 2.21, CI 1.42–3.43; *P* < 0.001; *P*_adj_ = 0.003), whereas controls more frequently reported jobs involving moderate physical activity (OR = 0.62, CI 0.45–0.86; *P* = 0.004; *P*_adj_ = 0.022) (Fig. 4C). In contrast, no group differences were observed in regular sports participation. The distribution of intensity of recreational activity or the frequency of exceptionally intense physical exertion within the 10 years preceding ALS symptom onset or survey completion was not different. Only contact sports were reported more frequently by controls, although this did not reach adjusted significance (OR = 0.60, CI 0.40–0.90; *P* = 0.014; *P*_adj_ = 0.06) (Fig. 4C).

Consumption habits in ALS were characterised by higher past but lower current consumption. Patients with ALS more frequently reported past consumption of caffeinated drinks (OR = 7.21, CI 3.27–15.89; *P* < 0.001; *P*_adj_ < 0.001), alcohol (OR = 2.25, CI 1.47–3.43; *P* < 0.001; *P*_adj_ = 0.002), and cigarettes or other tobacco products (OR = 1.64, CI 1.20–2.24; *P* = 0.002; *P*_adj_ = 0.011) (Fig. 4D). In contrast, controls were more likely to report current use of caffeine (OR = 0.43, CI 0.29–0.64; *P* < 0.001; *P*_adj_ < 0.001) and alcohol (OR = 0.56, CI 0.41–0.77; *P* < 0.001; *P*_adj_ = 0.003), and to have never smoked (OR = 0.61, CI 0.45–0.82; *P* = 0.001; *P*_adj_ = 0.008) (Fig. 4D; Supplementary Fig. S4). Quantities of consumption were not significantly different between groups (Supplementary Fig. S5).

### Diet and weight

Dietary patterns were largely comparable between groups, apart from calorie-restricted diets. Over the 10 years preceding symptom onset or survey completion, controls more frequently reported following a calorie-restricted diet (OR = 0.58, CI 0.39–0.85; *P* = 0.006; *P*_adj_ = 0.029). No significant differences were observed for fasting, adopting a vegetarian or vegan diet, or other dietary modifications (Supplementary Table S4).

Weight loss emerged before symptom onset and progressed over time in patients with ALS. At questionnaire completion, patients with ALS weighed significantly less than controls (OR = 0.63, CI 0.53–0.75; *P* < 0.001; *P*_adj_ < 0.001), corresponding to an average difference of 6.4 kg (Fig. 5A). While no significant group differences were observed at earlier time points, controls generally maintained stable or slightly increased weight over the preceding 10 years. In contrast, patients with ALS reported weight loss beginning 1–12 months prior to symptom onset, with mean loss of 5.6 kg from the year before onset to study completion. Additional weight loss was reported between symptom onset and diagnosis (1 kg), and from diagnosis to study completion (3.4 kg).

**Fig. 5:**
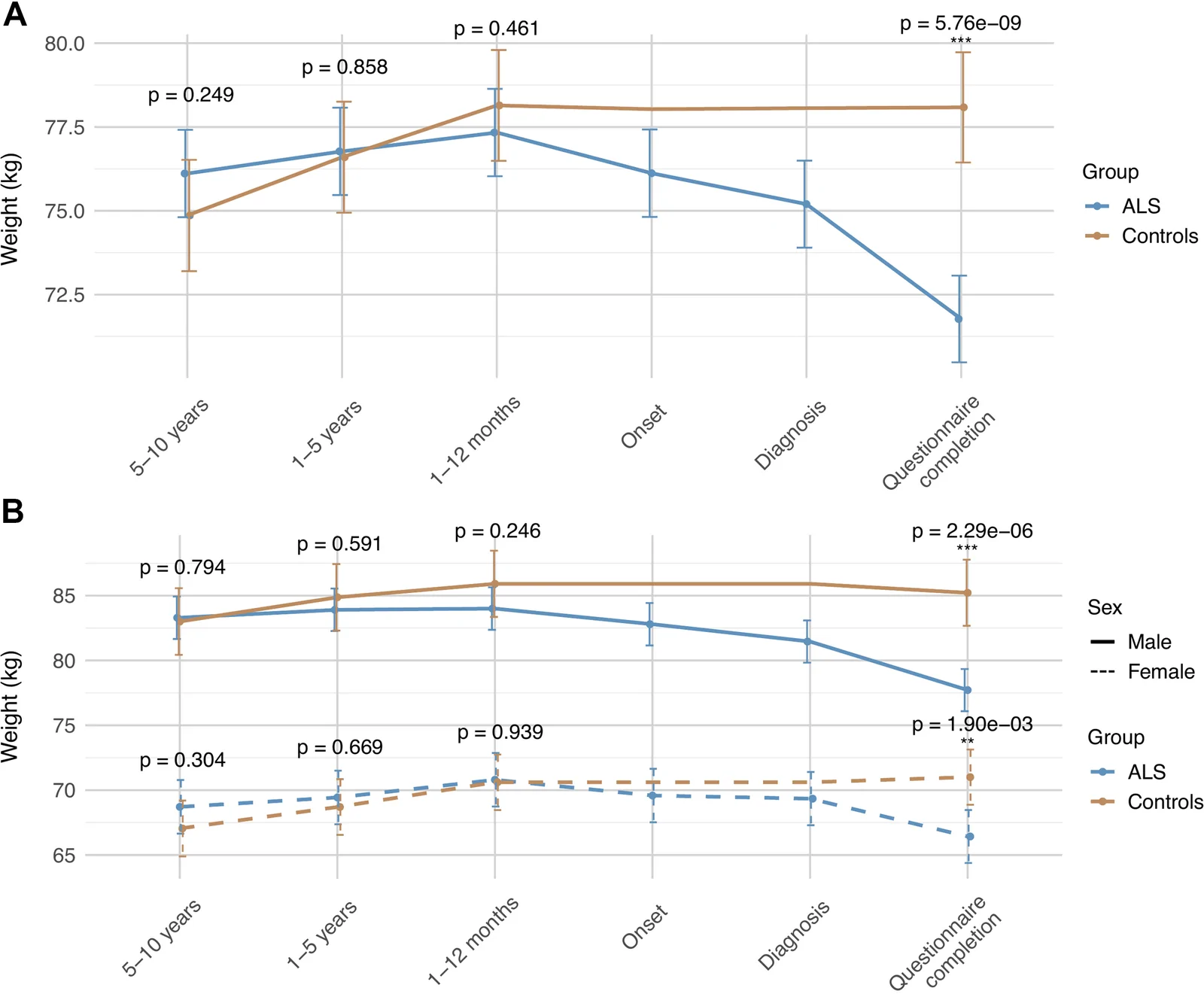
**Retrospective weight trajectories in patients with ALS and control participants. (A)** Weight data for patients with ALS (n = 469) and controls (n = 283) across six retrospective time periods, 5–10 years, 1–5 years, and 1–12 months before symptom onset (ALS) or time of questionnaire completion (controls), as well as at symptom onset (ALS), at diagnosis (ALS), and at questionnaire completion (both groups). Weight trajectories are shown as marginal means adjusted for sex, estimated from a linear mixed-effect model (LMM) with patient as a random effect and fixed effects for group, sex and time. **(B)** Weight data for patients with ALS and controls stratified by sex, across the same time periods as in **(A)**. Trajectories were obtained from an LMM with patient as a random effect and fixed effects of time and group. Solid lines represent male participants and dashed lines represent female participants. In both panels, lines indicate mean values with 95% confidence intervals. Statistical significance between groups was assessed using pairwise contrasts from the LMM, and is indicated as *P* < 0.05 (∗), *P* < 0.01 (∗∗), *P* < 0.001 (∗∗∗) (LMM, pairwise contrasts).

### Sensitivity analyses

Age-related sensitivity analyses were performed by excluding participants younger than 40 years (n = 21; 5 ALS and 16 controls) and, separately, within the subset with available age data (ALS n = 396; controls n = 148) (Supplementary Fig. S6; Supplementary Table S5). Overall, findings were broadly consistent with the primary analyses. Exclusion of participants younger than 40 years strengthened associations between current caffeine, alcohol, and cigarette consumption and ALS. In the age-available subset, associations for current caffeine and alcohol consumption changed direction, whereas associations for musculoskeletal disorders and past cigarette consumption no longer reached statistical significance. The principal findings regarding prodromal symptoms, healthcare utilisation, and weight trajectories remained unchanged.

### Subgroup analyses

Exploratory subgroup analyses by sex, site-of-onset, progression rate, disease duration, and age-at-onset revealed differences in reported prodromal symptom patterns between ALS subgroups and controls (Supplementary Fig. S7 and Tables S6–S11). For brevity, only ALS subgroup-versus-control comparisons are described below; results of within-subgroup comparisons for ALS clinical strata are provided in Supplementary Tables S8–S11. While trembling of the arms and legs was more frequently reported by both female and male patients with ALS (ORs >3; *P* < 0.05), excessive saliva was only reported more often by female patients (OR = 10.87, CI 1.39–85.06; *P* = 0.023). Trembling of arms and legs was more frequently reported by spinal-onset patients with ALS (OR = 3.33, CI 1.56–7.11; *P* = 0.002), remained consistently enriched across all progression-rate strata (ORs >3; all *P* < 0.05), was observed in both short- and long-duration patients (ORs >2; all *P* < 0.05), and showed its strongest association among early-onset patients (OR = 5.31, CI 2.44–11.53; *P* < 0.001). In contrast, bulbar-onset patients were significantly more likely to report excessive saliva (OR = 6.16, CI 2.37–16.06; *P* < 0.001), as well as, fast-progressors (OR = 5.11, CI 2.19–11.91; *P* < 0.001), patients with short-duration (OR = 3.90, CI 1.71–8.90; *P* = 0.001) and those with early-onset (OR = 2.54, CI 1.03–6.26; *P* = 0.042).

Prodromal neurology visits were more frequently reported in both female and male patients (ORs >1.2; all *P* < 0.05) and were significantly increased across all ALS subgroups (ORs >2; *P* < 0.001; Supplementary Fig. S7). Although speech therapy visits did not reach significance in the full cohort (*P* = 0.08), they were more frequently reported by female patients and across all ALS disease strata (ORs >2; all *P* < 0.05).

Overall musculoskeletal disorders and its subcategory herniated disc were the only pre-existing conditions showing significant associations across ALS subgroups. Musculoskeletal disorders were more frequently reported by spinal-onset patients (OR = 2.56, CI 1.67–3.93; *P* < 0.001), and remained significantly associated across all progression rates, disease-duration groups and both early- and late-onset patients (ORs >1.5; all *P* < 0.01). In contrast, herniated disc was more frequently reported only by male patients (OR = 2.21, CI 1.04–4.68; *P* = 0.038), spinal-onset patients (OR = 2.14, CI 1.25–3.67; *P* = 0.006), slow- and moderate-progressors (ORs > 2; *P* < 0.05), short- and long-duration patients (ORs > 1.5; *P* < 0.05), and those with early-onset disease (OR = 2.36, CI 1.31–4.26; *P* = 0.004). Adjustment for physically demanding occupations did not affect the significance of these associations (*P* < 0.05).

Being single was more frequently reported in both sexes and across most ALS subgroups (ORs >2; *P* < 0.05), with the exception of slow-progressors and early-onset patients. Having children was more common among female patients with ALS (OR = 1.83, CI 1.13–2.97; *P* = 0.015), spinal-onset patients (OR = 1.64, CI 1.14–2.36; *P* = 0.008), moderate-progressors (OR = 1.79, CI 1.10–2.90; *P* = 0.019), and patients with late-onset disease (OR = 1.69, CI 1.08–2.61; *P* = 0.019). Educational differences were more pronounced among females (Supplementary Fig. S8), who more frequently reported lower secondary school (OR = 2.20, CI 1.10–4.40; *P* = 0.026), and vocational or technical qualifications (OR = 2.05, CI 1.08–3.90; *P* = 0.028). Across disease strata, lower secondary education was also significantly more reported by spinal-onset patients (OR = 1.96, CI 1.23–3.10; *P* = 0.004), moderate- and fast-progressors (ORs > 1.5; *P* < 0.05), patients with both short- and long-duration (ORs > 1.5; *P* < 0.05), and those with late-onset (OR = 2.22, CI 1.30–3.77; *P* = 0.003). Vocational school attendance was more common in spinal-onset patients (OR = 1.66, CI 1.08–2.54; *P* = 0.020), slow-progressors (OR = 2.15, CI 1.29–3.59; *P* = 0.003) and long-duration groups (OR = 1.93, CI 1.21–3.08; *P* = 0.006). Male patients were more likely to report physically demanding occupations (OR = 1.80, CI 1.04–3.11; *P* = 0.035; Supplementary Fig. S9). Similar associations were observed among spinal-onset patients (OR = 2.59, CI 1.64–4.07; *P* < 0.001), slow- and fast-progressors (ORs > 2; *P* < 0.01), and patients with both short- and long-duration, as well as with both early- and late-onset ALS (ORs > 1.5; all *P* < 0.05). Past consumption habits showed marked sex-specific patterns: among females, past caffeine, alcohol, and smoking (ORs > 1.5; all *P* < 0.05) were more frequent in ALS, whereas among male patients only caffeine and alcohol use (ORs > 1.5; all *P* < 0.05) were increased (Supplementary Fig. S10). Across several disease strata, past consumption habits remained consistently and significantly enriched among patients with ALS, with the strongest association observed for caffeine (ORs > 2; all *P* < 0.05), followed by alcohol and cigarette consumption (ORs > 1; all *P* < 0.05; Supplementary Fig. S7). Only cigarette smoking was not significantly associated with ALS among moderate progressors (*P* = 0.12).

Detailed analyses of weight trajectories revealed that weight loss in ALS differed by sex, began years before symptom onset, and progressed over time. Across the study period, ALS males reported a mean loss of 5.6 kg and ALS females of 2.3 kg, whereas control males and females reported a gain of 2.2 kg and 4.0 kg, respectively. Across most time points up to 10 years before onset, male patients reported numerically lower weight than male controls, while no such group difference was observed among females (Fig. 5B). An ALS-only linear mixed-effects model showed a significant time-by-sex interaction during the pre-onset period (*P* = 0.009) (Fig. 5B), and sex-stratified models indicated greater weight loss only in male patients compared with controls (*P* < 0.001) (Supplementary Table S12). Reported weight loss continued after symptom onset, with males losing 1.3 kg from onset to diagnosis and a further 3.7 kg thereafter, and females losing 0.3 kg and 2.9 kg, respectively. At questionnaire completion, male patients weighed −7.4 kg relative to controls (OR = 0.58, CI 0.45–0.75; *P* < 0.001), and females −4.7 kg (OR = 0.69, CI 0.54–0.89; *P* = 0.004). Furthermore, disease subgroup analyses identified early weight differences across several clinical strata. At questionnaire completion, both bulbar- and spinal-onset (ORs < 1; *P* < 0.05), moderate- and fast-progressors (ORs < 1; *P* < 0.05), short-duration patients (OR = 0.76, CI 0.63–0.91; *P* = 0.003), and late-onset ALS (OR = 0.71, CI 0.58–0.87; *P* = 0.001) weighed less than controls (Supplementary Fig. S7 and Tables S8–S11).

## Discussion

While a prolonged prodromal phase is well recognised in other neurodegenerative diseases, it is less well defined in ALS. By the time diagnostic criteria are fulfilled, substantial motor neuron loss has already occurred.^23^ Identifying early disease-related changes is critical to enable earlier diagnosis, improve patient stratification, optimise clinical trial design, and ultimately improve therapeutic outcomes.^1^

In this study, patients with ALS reported a markedly higher burden of prodromal complaints in response to an open-ended question than controls, with symptoms predominantly affecting the neuromuscular system. Despite explicit instructions to report only symptoms preceding recognised ALS onset, 19% of patients described motor-related complaints, most commonly weakness and muscle cramps. Trembling of extremities was the most frequently reported prodromal complaint; however, this likely reflects subtle early motor involvement rather than a true non-motor feature. Together, these findings suggest that some patients retrospectively perceive neuromuscular changes before recognised ALS onset, potentially consistent with very early motor dysfunction. However, many of these symptoms likely represent very early symptomatic disease rather than a clearly separable prodromal phase, particularly as several findings clustered within the 1–12 months preceding recognised ALS onset.

Healthcare utilisation increased in the period preceding ALS onset, potentially suggesting an opportunity for early clinical recognition. Patients with ALS reported more frequent consultations with neurologists (35%) and speech therapists (8%), consistent with the possibility that some patients sought medical attention for early neurological symptoms prior to formal diagnosis.

Our findings can be interpreted within the emerging framework of mild motor impairment (MMI), a proposed prodromal state preceding clinically manifest ALS.^6^^,^^8^ MMI is characterised by subtle upper and/or lower motor neuron abnormalities, including clinical signs and electromyographic evidence of lower motor neuron denervation, that may precede the development of definite ALS by months or years. In the Pre-fALS study, a prospective cohort study of presymptomatic carriers of ALS-associated mutations, MMI was observed in more than 50% of individuals who later developed clinically manifest ALS.^24^ This is consistent with recent reports showing prodromal, primarily mild motor symptoms occurring within six months before recognised ALS onset in approximately one quarter of patients.^25^ Together with our findings, these observations suggest that MMI may also be relevant to predominantly sporadic ALS. However, these early symptoms are non-specific and should be interpreted cautiously, as they may also occur in other neurological or age-related conditions. Therefore, we support the view that ALS may evolve along a continuum in which prodromal and early clinical manifestations partially overlap, making a clear distinction between these stages inherently challenging, particularly in retrospective designs.

Sensory complaints represented the second most frequent prodromal feature, with pain being the most common. Sensory abnormalities, including paraesthesia, hypoesthesia, and features of sensory neuropathy, have been reported in 20–30% of patients with ALS, depending on the method of assessment.^26^^,^^27^ Pain can arise at any disease stage, and up to half of patients recall pain or sensory symptoms months to years before motor onset or diagnosis.^28^, ^29^, ^30^ Consistent with a recent questionnaire-based study reporting altered sensory perception and pain among the most common prodromal non-motor symptoms,^25^ our cohort reported predominantly back, limb, and muscle pain a median of two years before recognised ALS onset. These findings should be interpreted cautiously, as pain in prodromal ALS is likely heterogeneous and may partly arise secondary to other disease-related changes, such as muscle denervation, cramps, altered biomechanics, or musculoskeletal strain rather than primary sensory neuron pathology. This is supported by our observation, and previous reports, that MMI-associated features such as muscle cramps precede clinically manifest weakness, particularly in limb-onset disease,^25^^,^^31^ potentially reflecting early neuromuscular dysfunction before overt motor neuron degeneration becomes clinically apparent. The prominence of back and limb pain may also contribute to diagnostic uncertainty, particularly in patients initially evaluated for orthopaedic or spinal disorders. Recognising sensory- and pain-related complaints as a potential early feature of ALS warrants further investigation, as these symptoms may delay diagnosis and treatment initiation.^27^

We observed a higher incidence of prodromal musculoskeletal disorders, particularly herniated discs. This may partly reflect diagnostic bias, as early non-specific neuromuscular complaints often lead to spinal imaging and detection of incidental, but not necessarily causative disc pathology. However, because most herniated discs were reported 5 or more years before ALS onset, simple misattribution of early symptoms seems less likely. Nevertheless, orthopaedic misdiagnosis remains common in ALS, with some patients undergoing inappropriate spinal surgery.^32^^,^^33^ Alternatively, disc prolapse itself may contribute to focal neural injury that could initiate or accelerate neurodegeneration,^34^ although this cannot be addressed by the present study.

In contrast to the open-ended prodromal complaints reports, and unlike findings in other neurodegenerative disorders, relatively few specific prodromal non-motor changes were identified in ALS when assessed using predefined symptom categories. This may reflect systematic underreporting, as patients retrospectively prioritise salient motor deficits while subtle non-motor changes fade from memory leading to recall bias. Supporting this interpretation, patients with ALS may report normal subjective sleep despite marked alterations in sleep architecture,^35^ potentially related to involvement of von Economo neurons in the anterior cingulate cortex, which are critical for subjective judgement.^36^ Moreover, since impaired self-awareness or agnosia is common in frontotemporal dementia (FTD),^37^, ^38^, ^39^ and (mild) cognitive/behavioural impairments occur in around 50% of patients with ALS,^40^^,^^41^ reduced awareness of symptoms cannot be excluded.

As many controls were spouses or relatives of patients with ALS, the elevated burden of prodromal non-motor changes in controls including sensory, sleep- and fatigue-related, and behavioural symptoms, as well as increased healthcare utilisation outside neurology and higher rates of infections, may partly reflect caregiving-related psychosocial stress previously reported among caregivers^42^^,^^43^ and increased healthcare engagement. By contrast, patients with ALS, who have been described as exhibiting high agreeableness,^44^ may be less inclined to modify life circumstances despite emerging difficulties, potentially contributing to lower reporting of such non-motor changes.

Patients with ALS, particularly females, more frequently reported lower educational attainment, alongside a higher prevalence of physically demanding occupations, especially among males. These findings align with prior evidence linking manual labour and lower socioeconomic status to increased ALS risk.^45^, ^46^, ^47^ In contrast, lifetime engagement in recreational sports was similar between groups, suggesting that occupational exposures, characterised by sustained intensity and cumulative burden, may be more relevant than leisure activity.^48^^,^^49^ Potential exposure to environmental toxins, more common in physically demanding occupations, including heavy metals, pesticides, or solvents, also has been implicated in ALS pathogenesis.^47^^,^^50^

Patients with ALS reported higher past but lower current use of caffeine, alcohol, and cigarettes, whereas controls more often reported ongoing consumption, likely reflecting lifestyle changes following symptom onset or diagnosis. While overall consumption habit exposure was similar, patients with ALS showed a trend towards higher high-level smoking in the past, consistent with epidemiological evidence reporting associations between smoking and ALS risk.^51^^,^^52^ In contrast, evidence for alcohol remains inconsistent.^53^, ^54^, ^55^ While caffeine has been suggested to exert neuroprotective effects in Parkinson’s disease even at relatively low intake,^56^ current and prospective studies show no association between pre-diagnostic caffeine intake and ALS risk,^57^ with experimental data suggesting potential harm in *SOD1* mouse models.^58^

Controls more frequently reported calorie-restricted diets, possibly reflecting a reduced need for dietary restriction in ALS due to disease-related weight loss. Consistent with previous studies, patients with ALS showed substantial early weight loss,^10^^,^^59^ a key non-motor feature associated with poorer prognosis.^11^ In our cohort, weight loss was reported 1–12 months before symptom onset and continued thereafter. Although weight loss may partly result from reduced nutritional intake secondary to dysphagia,^10^ loss of appetite, reduced caloric intake, and weight loss have also been documented in ALS independently of dysphagia.^60^ The mechanisms underlying weight loss in ALS are likely complex and multifactorial, involving interactions among dysphagia, appetite loss, reduced energy intake, altered hypothalamic regulation, changes in metabolic demand, muscle wasting, behavioural factors, and psychosocial influences such as anxiety and depression during the period preceding diagnosis.^12^^,^^61^, ^62^, ^63^, ^64^ Altogether, these findings highlight the potential value of early and sustained nutritional assessment and management in ALS.

Subgroup analyses revealed distinct patterns across clinical subgroups, suggesting that the prodromal phase may not be uniform but instead differ according to clinical phenotype. Several associations, most notably increased prodromal neurology consultations and, more variably, higher past caffeine, alcohol, and cigarette consumption, were observed across sex, site-of-onset, progression, disease-duration, and age-at-onset strata, suggesting that these may represent general features of the ALS prodrome rather than phenotype-specific markers. By contrast, symptom-related findings showed greater clinical specificity. Excessive saliva was particularly prominent in females, bulbar-onset patients, and fast-progressors, and was accompanied by increased pre-diagnostic speech therapy visits, likely reflecting the higher prevalence of bulbar-onset ALS among females and the established association between bulbar involvement and more aggressive disease.^65^^,^^66^ Interestingly, prodromal trembling of the extremities showed its strongest association in early-onset ALS. Although this finding requires replication, it raises the possibility that the presentation of MMI may vary according to age at disease onset. Whether this reflects age-related differences in disease biology, symptom perception, or healthcare-seeking behaviour remains unclear. Musculoskeletal disorders and herniated discs were more frequent in spinal-onset ALS, with herniated discs also more common among males. The epidemiology of disc herniation is inconsistent, with some surgical cohorts reporting a slight male predominance,^67^ while others describe higher rates in women.^68^ In our cohort, males and spinal-onset patients were also more likely to have held physically demanding jobs. Although lumbar disc herniation has been linked to manual labour,^68^^,^^69^ intensive physical work did not explain this association in our cohort, suggesting additional contributions from chronic spinal microtrauma, sex-specific biology, or delayed healthcare-seeking in men. Consistent with prior reports of greater weight loss during disease progression,^70^ we found that pre-morbid weight loss was more pronounced in men. Together with emerging evidence of greater molecular alterations in male ALS,^71^ these sex-specific patterns may be associated with differential motor neuron vulnerability, providing a hypothesis-generating framework for exploring sex differences in ALS prevalence and clinical phenotype.

This study has several limitations. Recall bias is a major concern, particularly for vague or nonspecific symptoms. Because data were collected retrospectively after ALS diagnosis, patients may have reinterpreted prior bodily changes and other health-related experiences in light of their disease, potentially influencing retrospective reporting and leading to overreporting of prodromal symptoms and related health events. Temporal relationships cannot be established reliably, and some associations may reflect reverse causality rather than true prodromal changes. Patients and controls also differed in their temporal reference points (symptom onset/diagnosis versus questionnaire completion), an inherent limitation of retrospective prodromal case–control studies that complicates temporal comparisons. Reliance on self-reported questionnaires may have reduced accuracy, particularly in patients with mild cognitive impairment or proxy-assisted completion. The absence of clinical, biomarker, neurophysiological, or imaging measures precluded independent validation of reported prodromal features, highlighting the need for prospective multimodal studies.^72^ Controls were primarily relatives, partners, and caregivers, potentially including selection bias, shared environmental exposures, and age-related confounding, thereby limiting generalisability. Future studies should therefore include more representative population-based control groups. Residual confounding from unmeasured factors cannot be excluded. Incomplete age data precluded adjustment in the primary analyses. Nevertheless, age-related sensitivity analyses yielded broadly consistent results, supporting the robustness of the main findings. Finally, despite the relatively large cohort, multiple testing, sparse responses, and subgroup analyses increase the risk of false-positive findings and limited power for smaller effects. In particular, borderline associations that did not survive FDR correction should be interpreted cautiously and require replication in independent cohorts.

Our findings suggest that a subset of patients with ALS retrospectively report a prodromal phase during which neurological and/or speech therapy services were already engaged, highlighting potential opportunities for earlier recognition. By providing evidence consistent with prodromal mild motor impairment in a large, multicentre, predominantly sporadic ALS cohort, this study provides a foundation for refining clinical frameworks aimed at identifying ALS at earlier stages. Moreover, the observation that weight loss may emerge closer to motor symptom onset raises the possibility that early weight changes could represent an underrecognised feature of prodromal ALS and may warrant further prospective investigation in the context of future risk stratification and trial enrichment strategies.

## Contributors

IC, AGa, MPM, PL contributed equally to this work. IC and PL conceived and designed the study. AGa conducted the statistical analyses and performed the visualisations, with supervision by MPM. IC, AGa, PL, and MPM directly accessed and verified the underlying data and contributed to data interpretation. All authors contributed to data acquisition, critically revised the manuscript, and approved the final version for publication.

## Data sharing statement

Analysis and scripts are publicly available at https://github.com/MendenLab/EARLY-questionnaire. Original data are available on reasonable request from the corresponding authors.

## Declaration of interests

IC reports grants from the Hertie Network of Excellence in Clinical Neuroscience and the TUM Clinician Scientist Program KKF TUM H-26.

AGa reports funding from the Bundesministerium für Bildung und Forschung in the scope of the EU Joint Programme—Neurodegenerative Disease Research (JPND) through the BMBF (01ED2204A, 01ED2204B) under the aegis of JPND; and support for attending meetings and/or travel from a JPND grant for attendance at ENCALS meetings in 2025 and 2026.

FK reports funding from TUM Klinikum for implementation of the study registry/data collection, with payments made to Bitcare GmbH, which is owned by FK; and stock or stock options in Bitcare GmbH.

BB reports consulting fees from Roche; payment or honoraria from Biogen and Roche; support for attending meetings and/or travel from ITF Pharma GmbH; and other financial or non-financial interests through support from the PRACTIS Clinician Scientist Program, funded by Hannover Medical School and the Deutsche Forschungsgemeinschaft (DFG; ME 3696/3).

MS reports grants from the GBS|CIDP Foundation Elevation Award 2024; consulting fees from Takeda Pharmaceutical; support for attending meetings and/or travel from Takeda Pharmaceutical; and participation on a Data Safety Monitoring Board or Advisory Board for Takeda Pharmaceutical. These relationships are not related to the current manuscript and/or its topics.

MD reports consulting fees from Hoffmann-La Roche; payment or honoraria from AstraZeneca, Hoffmann-La Roche, and Sanofi; and participation on a Data Safety Monitoring Board or Advisory Board for Amicus, Biogen, Hoffmann-La Roche, and Sanofi.

DB reports grants from the Else Kröner Foundation, German Society for Muscle Disorders, Thyssen Foundation, Dürr Foundation, and Frick Foundation; payment or honoraria from Biogen; support for attending meetings and/or travel from Biogen; participation on an Advisory Board for Biogen; and stock or stock options in Ionis Pharmaceuticals.

SKB reports grants from the Bavarian State Ministry of Health; consulting fees from ITF Pharma; and support for attending meetings and/or travel from Zambon.

SL reports grants from the Bavarian Ministry of Health; payment or honoraria from STADA, BIAL, and Boehringer; and support for attending meetings and/or travel from STADA.

AH reports grants from Hermann and Lilly Schilling Stiftung, BMBF/VDI, and DFG; royalties or licences from Kohlhammer and Elsevier; and consulting fees from Zambon and ITF Pharma.

TH reports consulting fees from Biogen; payment or honoraria from Biogen; and participation on an Advisory Board for Biogen.

JWo reports being a member of the steering committee of the German Motor Neuron Disease Network.

MR reports grants from the Förderverein für HSP-Forschung e.V. and the Bundesministerium für Forschung, Technologie und Raumfahrt; payment or honoraria from Zambon GmbH, Bial GmbH, DGN e.V., Orphalan GmbH, Ever Pharma GmbH, and AbbVie GmbH; and support for attending meetings and/or travel from Merz Pharma GmbH, the National Institutes of Health, Ipsen GmbH, AbbVie GmbH, and Zambon GmbH.

RG reports grants from the Bundesministerium für Bildung und Forschung and the Deutsche Gesellschaft für Muskelkranke, outside of the submitted work; and payment or honoraria from Biogen, Zambon, and Roche, outside of the submitted work.

AM reports payment or honoraria from AstraZeneca GmbH, Zambon Pharma GmbH, and Roche Pharma AG; support for attending meetings and/or travel from Ipsen Pharma GmbH and ITF Pharma; and participation on a Data Safety Monitoring Board or Advisory Board for Novartis Pharma GmbH and Roche Pharma AG.

DZ reports support for attending meetings and/or travel from ITF Pharma, Munich.

CN reports payment or honoraria from Amgen, Amicus, and Takeda; support for attending meetings and/or travel from Amgen and Amicus; participation on a Data Safety Monitoring Board or Advisory Board for Alexion, Amicus, argenx, Biogen, F. Hoffmann-La Roche, Novartis, and Takeda; and being a member of EAN panels for neurophysiology, neuromuscular junction disorders, ALS, and FTD.

MWei reports consulting fees from Akcea Therapeutics, Alnylam Pharmaceuticals, Biogen, F. Hoffmann-La Roche, and Swedish Orphan Biovitrum AB; payment or honoraria from Akcea Therapeutics, Alnylam Pharmaceuticals, AstraZeneca, and Biogen; support for attending meetings and/or travel from Akcea Therapeutics, Alnylam Pharmaceuticals, AstraZeneca, BridgeBio, Ionis, and Pfizer; and participation on an Advisory Board for Akcea Therapeutics, Alnylam Pharmaceuticals, Biogen, F. Hoffmann-La Roche, Novartis, Novo Nordisk, Pfizer, Purpose Pharma, and Swedish Orphan Biovitrum AB.

SP reports grants from the German Neuromuscular Society (DGM), German Israeli Foundation for Scientific Research and Development (GIF), Neurodegenerative Research Inc, Karlheinz Hartmann Foundation, and Petermax Müller Foundation; consulting fees from Biogen GmbH, Italfarmaco, and Amylyx; and payment or honoraria from Biogen, Italfarmaco, Amylyx, Zambon, Roche, and Novartis.

MPM reports grants from GSK, Roche, and AstraZeneca, unrelated to the submitted work; consulting fees from Merck Sharp & Dohme and McKinsey & Company; payment or honoraria from the CAS in Personalised Medicine at the University of Lausanne; and patents for WO/2025/021719A1 and WO/2025/051923.

PL reports grants from the Bundesministerium für Bildung und Forschung, the Deutsche Forschungsgemeinschaft, LifeArc, the Longitude Prize/Nesta, the Michael J. Fox Foundation for Parkinson’s Disease, and JPND; consulting fees from AbbVie, Amylyx, Bial, Crucible Therapeutics, Desitin, ITF Pharma, Novartis, Stadapharm, Raya Therapeutic, Woolsey Pharmaceuticals, Trace Neuroscience, and Zambon; and being a co-inventor on a patent for the use of fasudil in amyotrophic lateral sclerosis (EP 2825175 B1, US 9,980,972 B2).

The remaining authors report no conflicts of interest.
