# Supplementary material for: EARLY-ALS: a multicentre study on prodromal amyotrophic lateral sclerosis
Source: eBioMedicine. 2026 Oct 9;133:106505. doi: 10.1016/j.ebiom.2026.106505 (PMC13677275; doi:10.1016/j.ebiom.2026.106505)
Supplement: Supplementary file 1 [file mmc1.docx]

**Supplementary Materials**

Table of Contents

[Supplementary Methods	2](#_Toc237345827)

[Supplementary Figures	4](#_Toc237345828)

[Fig. S1: Participant flow diagram.	4](#_Toc237345829)

[Fig. S2: Heatmaps before and after manual imputation.	5](#_Toc237345830)

[Fig. S3: Time distribution of neuro/motor and sensory/vestibular symptoms in patients with ALS (n=106).	6](#_Toc237345831)

[Fig. S4: Consumption habits among patients with ALS (n=475) and control (n=285) participants.	7](#_Toc237345832)

[Fig. S5: Quantification of consumption habits among patients with ALS and control participants.	8](#_Toc237345833)

[Fig. S6: Sensitivity analyses of age against sex-adjusted analyses of the full sample.	9](#_Toc237345834)

[Fig. S7: Response patterns across ALS disease strata.	10](#_Toc237345835)

[Fig. S8: Highest educational and professional qualifications among patients with ALS and control participants, stratified by sex.	11](#_Toc237345836)

[Fig. S9: Occupational physical activity and regular sports participation among patients with ALS and control participants, stratified by sex.	12](#_Toc237345837)

[Fig. S10: Consumption habits among patients with ALS and control participants, stratified by sex.	13](#_Toc237345838)

# **Supplementary Methods**

**Development and Implementation of the EARLY-ALS Questionnaire**

The EARLY-ALS questionnaire was specifically developed for this multicentre study to systematically assess prodromal symptoms, healthcare utilisation, comorbidities, environmental exposures, lifestyle factors, and sociobiographical events in the years preceding ALS symptom onset. As no established instrument was available that comprehensively captured these domains in predominantly sporadic ALS, the questionnaire was designed de novo by the study investigators based on clinical expertise, experience in ALS diagnostics and early neurodegenerative disease manifestations, and review of the existing literature on prodromal ALS, mild motor impairment (MMI), non-motor symptoms, and ALS-associated environmental risk factors and lifestyle-related factors.

The questionnaire was implemented using the open-source survey platform LimeSurvey and delivered digitally via secure HTTPS access. Participants received unique personal registration tokens during routine clinic visits, allowing pseudonymised linkage between patients with ALS and matched controls. Responses and survey data were stored on a secure institutional server. Separate survey versions were developed for patients with ALS and controls, with conceptually corresponding items adapted to the respective participant group.

Before survey initiation, participants received written instructions explaining the study purpose, the approximate completion time (20–30 minutes), and contact information for the responsible study physician in case questions arose during participation. Given the retrospective questionnaire-based design, all responses relied on participant recall. Participants were informed that temporal information could be estimated approximately when exact dates were uncertain and that approximate years were acceptable for retrospective reporting. The questionnaire further provided a standardised definition of “disease onset” as the occurrence of the first symptom attributable to the motor neuron disease (e.g., muscle weakness, gait disturbance, or swallowing difficulties). For many items, patients were explicitly instructed to report changes occurring before recognised disease onset and not attributable to ALS or another known medical condition.

The survey comprised seven sections: 1) demographics; 2) disease-related aspects including self-reported ALS phenotype, genetic testing status and results, including the presence or absence of ALS-associated mutations, and medication; 3) prodromal complaints and early non-motor changes across organ systems; 4) healthcare system interactions; 5) comorbidities; 6) lifestyle changes including occupation, physical activity, diet, and social life; and 7) the self-assessed ALS Functional Rating Scale-Revised (ALSFRS-R). Participants self-reported their sex using the categories female and male; no distinction was made between biological sex, sex assigned at birth, or gender identity.

Most survey items were closed-ended with predefined response categories. At the end of relevant sections, free-text “Other” fields were additionally provided to allow participants to report individual experiences not captured by predefined options. Importantly, the third section began with an open-ended question inviting participants to report any noticeable bodily or behavioural changes that had occurred before recognised ALS onset and could not be explained by pre-existing comorbidities (“prodromal complaints”). This was followed by a structured, closed-ended assessment of prodromal non-motor changes across predefined symptom categories. Most questions referred to the 10-year period prior to ALS symptom onset. For controls, the reference period was defined as the 10 years preceding survey completion. In both groups, the reference period was subdivided into four intervals: 5–10 years, 1–5 years, 1–12 months, and less than 1 month. For free-text prodromal responses and for the comorbidities section, participants indicated the timing of each complaint, which was subsequently assigned to the predefined time intervals to ensure consistent temporal categorisation. Responses reporting symptom or condition onset after ALS symptom onset were excluded.

The questionnaire employed adaptive conditional logic to reduce unnecessary burden and improve usability. Follow-up questions were displayed only when relevant based on previous responses. For example, selecting a broad symptom category such as sensory perception changes triggered additional questions regarding specific symptoms (e.g., reduced sense of smell). Similar conditional branching was applied throughout the survey, including healthcare utilisation and lifestyle sections. To facilitate accurate completion, explanatory examples and contextual guidance were provided repeatedly throughout the questionnaire. Participants were explicitly informed that some questions might not apply to their individual situation and that such items should be left unanswered.

Because many patients with ALS may experience motor impairment affecting computer use, proxy-assisted completion was permitted and was explained by the recruiting physician during the study information and consent discussion. In this context, “proxy-assisted” referred to practical support by another person (e.g., partner, relative, or caregiver) during questionnaire completion, such as reading questions aloud, navigating the digital interface, or entering responses dictated by the participant. Responses were intended to reflect the participant’s own experiences and judgments whenever possible. However, given the retrospective self-reported design, some influence of assisting individuals on response interpretation or reporting cannot be excluded. Similarly, mild cognitive or behavioural impairment associated with ALS may have affected recall accuracy or symptom awareness in some participants.

Due to the comprehensive nature of the questionnaire and the large number of conditionally displayed items, participant burden and response fatigue cannot be excluded. To mitigate fatigue-related effects, participants could complete the survey in multiple sessions. The survey was designed to be completed flexibly according to participant preference and physical ability, with the option to pause and resume the online questionnaire at a later time point. Furthermore, the questionnaire employed adaptive branching logic, concise response formats, explanatory examples, and optional omission of non-applicable items. Nevertheless, incomplete responses occurred in a subset of participants and represent an inherent limitation of the study design.

# **Supplementary Figures**

## **Fig. S1: Participant flow diagram.**

Summary of participant flow. A total of 818 participants were recruited, including 513 patients with ALS and 305 control participants. Following exclusion of 38 patients with ALS and 20 controls due to incomplete questionnaire completion, 475 patients with ALS and 285 controls remained for analysis. The final analytical cohort comprised 760 participants.

## **Fig. S2: Heatmaps before and after manual imputation.**

**(A)** Overview of responses to the original questionnaire. Dark grey cells represent provided answers, where white cells indicate questions that were left unanswered. Participants (ALS: n=475; controls: n=285) were instructed to leave items blank if they were not applicable or if they were unsure, resulting in a considerable number of non-responses in the initial dataset. **(B)** Overview after manual imputation. For selected binary items and questions about the number of visits to specific healthcare specialists (continuous), missing responses were interpreted as "no" or zero visits, under the assumption that non-response indicated non-occurrence during the specified time frame. In both panels, questions are categorised as binary, categorical or continuous, and the percentage of responses (i.e., answer rate; %) is shown on the left-hand side of each heatmap. Group affiliation (ALS or controls) is indicated in the upper annotation of each heatmap.

## **Fig. S3: Time distribution of neuro/motor and sensory/vestibular symptoms in patients with ALS (n=106).**

Density distributions of reported neuro/motor and sensory/vestibular symptoms, quantified in years relative to the date of ALS symptom onset (Onset). Only responses for symptoms spanning the pre-onset period were analysed and visualised. The density of responses is coloured by symptom type.

## **Fig. S4: Consumption habits among patients with ALS (n=475) and control (n=285) participants.**

Bar graphs displaying patterns of consumption habits, including **(A)** caffeine, **(B)** alcohol, and **(C)** cigarette consumption, categorised as current use, past use, or never. Participants could select only one response category for each habit. Missing values for consumption habits were categorised as not available (n.a.). Significance was assessed using univariate logistic regression analyses stratified by sex and is indicated as *P <* 0·05 (*), *P <* 0·01 (**), *P <* 0·001 (***). Bars represent percentages; no error bars are displayed.

## **Fig. S5: Quantification of consumption habits among patients with ALS and control participants.**

Bar graphs illustrate the quantities of **(A)** caffeine, **(B)** alcohol, and **(C)** cigarette consumption. Only participants who reported current or past consumption habits were included in these distributions. For caffeine and alcohol, participants reported their typical intake based on predefined consumption categories. Specifically, **(A)** Caffeine consumption was classified as low (1–2 cups of coffee/day), moderate (3–5 cups/day), and high (>5 cups/day). Participants were instructed that one cup of black coffee corresponds approximately to the caffeine content of one liter of cola or one can of an energy drink. A total of 409 ALS and 249 controls reported caffeine consumption. **(B)** Alcohol consumption was classified based on typical daily intake, with a standard drink defined as 0·25 L beer, 0·1 L wine or sparkling wine, or 0·04 L spirits; low consumption was ≤1 drink/day for women or ≤2 drinks/day for men (≤5 days/week), and high consumption was above these thresholds. A total of 229 ALS and 142 controls reported alcohol consumption. **(C)** For smoking, participants indicated the number of cigarettes smoked per day. To standardise reporting, smoking quantities were grouped into three categories: low (1–6 cigarettes/day), moderate (7–14 cigarettes/day), and high (>14 cigarettes/day), based on the reported amounts. A total of 253 ALS and 132 controls reported cigarette consumption. The frequencies for each consumption habit was calculated based on all participants who reported current or past use, respectively. Missing values were categorised as not available (n.a.).

## **Fig. S6: Sensitivity analyses of age against sex-adjusted analyses of the full sample.**

**(A)** Age distribution of patients with ALS (n=396) and controls (n=148) with available age data. The vertical line indicates the age threshold of 40 years used for younger participant exclusion.

**(B)** Comparison of sex-adjusted analyses in the full sample versus the full sample excluding participants younger than 40 years (n=21; 5 ALS and 16 controls). **(C)** Comparison of sex-adjusted analyses in the full sample (ALS: n=475; controls: n=285) versus sex- and age-adjusted analyses restricted to participants with available age data (ALS: n=396; controls: n=148). **(D)** Comparison of the same cohorts as in (C), with both analyses adjusted for sex only.

In panels **(B)**, **(C)** and **(D)**, each point represents a questionnaire item, plotted as signed log p-values defined as sign(odds-ratio) x (-log(p-value)), simultaneously capturing the direction and magnitude of each association. The p-values were estimated based on univariate logistic regression analyses (Wald test). Points along the diagonal indicate concordant results between the models. Points to the right of the axis indicate associations more frequent in patients with ALS, and points to the left indicate associations more frequent in controls. Colour coding indicates significance status: grey, non-significant in both models; orange, significant in both models; blue, significant in the primary analysis only; green, significant in the sensitivity analysis only. Dashed vertical and horizontal lines at ±log(0.05), to separate significant and non-significant results. Labelled points represent the most notable associations.

## **Fig. S7: Response patterns across ALS disease strata.**

Heatmap displaying odds ratios (ORs) from multinomial logistic regression models for questionnaire items significantly enriched in patients with ALS in the primary analysis. Each row represents a questionnaire item, and each column an ALS subgroup compared with controls. The first column shows the original sex-adjusted estimate from the primary analysis (475 patients with ALS vs 285 controls), followed by sex-stratified analyses for females (180 patients with ALS vs 168 controls) and males (289 patients with ALS vs 114 controls). The remaining columns show sex-adjusted subgroup analyses stratified by site-of-onset (bulbar-onset ALS, n = 75; spinal-onset ALS, n = 378), progression rate (slow-progressors, n = 153; moderate-progressors, n = 146; fast-progressors, n = 154), disease duration (short-duration, n = 228; long-duration, n = 225), and age-at-onset (early-onset, n = 191; late-onset, n = 185). All subgroup models used controls as the reference category. Colour intensity reflects the magnitude of the log(OR), with red indicating higher odds and blue indicating lower odds relative to controls.

Significance is indicated as *P <* 0·05 (*), *P <* 0·01 (**), *P <* 0·001 (***). For non-significant comparisons, the raw p-value is depicted.

## **Fig. S8: Highest educational and professional qualifications among patients with ALS** **and control participants, stratified by sex.**

Bar graphs show the distribution of **(A)** educational degrees and **(B)** professional qualifications for female (ALS: n=180; controls: n=168) and male (ALS: n=289; controls: n=114) participants separately.

Missing educational and professional qualifications of participants were categorised as not available (n.a.). Significance was assessed using univariate logistic regression analyses stratified by sex and is indicated as *P <* 0·05 (*), *P <* 0·01 (**), *P <* 0·001 (***).

## **Fig. S9: Occupational physical activity and regular sports participation among patients with ALS** **and control participants, stratified by sex.**

Bar graphs displaying the distribution of **(A)** physical activity related to occupation, categorised as low, moderate, or high intensity, and **(B)** regular sports participation, also classified into low, moderate, or high intensity, presented separately for female (ALS: n=180; controls: n=168) and male (ALS: n=289; controls: n=114) participants.
Missing physical activity in professional and sport activities of participants were categorised as not available (n.a.). Significance was assessed using univariate logistic regression analyses stratified by sex and is indicated as *P <* 0·05 (*), *P <* 0·01 (**), *P <* 0·001 (***).

## **Fig. S10: Consumption habits among patients with ALS** **and control participants, stratified by sex.**

Bar graphs displaying patterns of consumption habits, including **(A)** caffeine, **(B)** alcohol, and **(C)** cigarette consumption, categorised as current use, past use, or never. Data are presented separately for female (ALS: n=180; controls: n=168) and male (ALS: n=289; controls: n=114) participants.

Missing consumption habits of participants were categorised as not available (n.a.). Significance was assessed using univariate logistic regression analyses stratified by sex and is indicated as *P <* 0·05 (*), *P <* 0·01 (**), *P <* 0·001 (***).
